# Linking Proteins to Signaling Pathways for Experiment Design and Evaluation

**DOI:** 10.1371/journal.pone.0036202

**Published:** 2012-04-27

**Authors:** Illés J. Farkas, Ádám Szántó-Várnagy, Tamás Korcsmáros

**Affiliations:** 1 Statistical and Biological Physics Research Group, Hungarian Academy of Sciences, Budapest, Hungary; 2 Department of Biological Physics, Eötvös Loránd University, Budapest, Hungary; 3 Department of Genetics, Eötvös Loránd University, Budapest, Hungary; Koc University, Turkey

## Abstract

Biomedical experimental work often focuses on altering the functions of selected proteins. These changes can hit signaling pathways, and can therefore unexpectedly and non-specifically affect cellular processes. We propose PathwayLinker, an online tool that can provide a first estimate of the possible signaling effects of such changes, e.g., drug or microRNA treatments. PathwayLinker minimizes the users' efforts by integrating protein-protein interaction and signaling pathway data from several sources with statistical significance tests and clear visualization. We demonstrate through three case studies that the developed tool can point out unexpected signaling bias in normal laboratory experiments and identify likely novel signaling proteins among the interactors of known drug targets. In our first case study we show that knockdown of the *Caenorhabditis elegans* gene *cdc-25.1* (meant to avoid progeny) may globally affect the signaling system and unexpectedly bias experiments. In the second case study we evaluate the loss-of-function phenotypes of a less known *C. elegans* gene to predict its function. In the third case study we analyze GJA1, an anti-cancer drug target protein in human, and predict for this protein novel signaling pathway memberships, which may be sources of side effects. Compared to similar services, a major advantage of PathwayLinker is that it drastically reduces the necessary amount of manual literature searches and can be used without a computational background. PathwayLinker is available at http://PathwayLinker.org. Detailed documentation and source code are available at the website.

## Introduction

Signaling pathways regulate several cellular processes, including cell growth, differentiation, stress response and adaptation [1], [2]. In addition, signaling pathways control cell-cell communication and organism-level processes in the immune and hormone systems. Malfunctions of signaling pathways may lead to ‘systems diseases’ [3], for example, cancer or diabetes. Accordingly, perturbations of signaling proteins can non-specifically affect many cellular processes. Altering the functions of selected genes or proteins is a common procedure in biomedical research and can lead to the discovery of novel molecular functions and organism-level characteristics. Unfortunately, these modifications often induce unexpected and unwanted changes in the normal functions of signaling pathways. The first step towards quantifying the effects of these signaling changes is the identification of known signaling components among the modified proteins and their interacting partners. Currently, most research laboratories perform this step either by (i) time-consuming manual literature searches (requiring expertise with signaling pathways) or (ii) a semi-automatic scan of databases (requiring advanced programming skills). PathwayLinker is meant to be an efficient alternative to both resource-intensive approaches. It identifies the interactions linking the queried protein(s) to signaling pathways by applying the interaction and pathway data types selected by the user. PathwayLinker allows experimentalists and computational biologists to quickly and systematically obtain first estimates of the possible signaling effects of modifying single proteins or selected groups of proteins (e.g., by microRNAs or as drug targets).

## Materials and Methods

We integrated physical and genetic interaction data and signaling pathway membership data for the nematode *Caenorhabditis elegans*, the fruit fly *Drosophila melanogaster*, and humans. For the interactions, we used small-scale and high-throughput physical interactions from BioGRID (version 3.0.64) and STRING (version 8.3), small-scale physical, high-throughput physical, and genetic interactions from the Worm Interactome (version 8) and DroID (version 2010_08), and small-scale physical interactions of human proteins from HPRD (version 5) [4]–[8]. Signaling pathway reference data was compiled by integrating data from the pathway databases KEGG (version 53.0), Reactome (version 33), and SignaLink (version 1.0) [9]–[11]. In each case we mapped gene/protein names to UniProt primary accessions, human-readable protein names and database-specific identifiers (Flybase ID, Wormbase ID, Ensembl gene and protein ID, etc.) with the programmable online services of UniProt [12].

PathwayLinker speeds up searches and protein identifier (ID) conversions by saving all new ID mapping results locally and accessing these at subsequent ID mapping requests. Analysis subroutines identify interactors and pathway member proteins among them and compute statistical tests. Cytoscape Web [13] a jQueryUI are used for visualization and the user interface.

To estimate the signaling effect of the queried proteins and their first neighbor interactors, PathwayLinker tests for each signaling pathway whether it is statistically significantly overrepresented among the queried proteins. First, the queried proteins and their first neighbor interactors are listed. Next, for each signaling pathway, *S*, PathwayLinker counts how many (*N*) of these proteins function in *S*. Last, the p-value of the overrepresentation of each pathway is computed by finding the probability of observing at least *N* such proteins (i.e., a hypergeometric distribution is applied). For details, see http://pathwaylinker.org/pathway_score.

## Results

### The workflow

**Figure 1:**
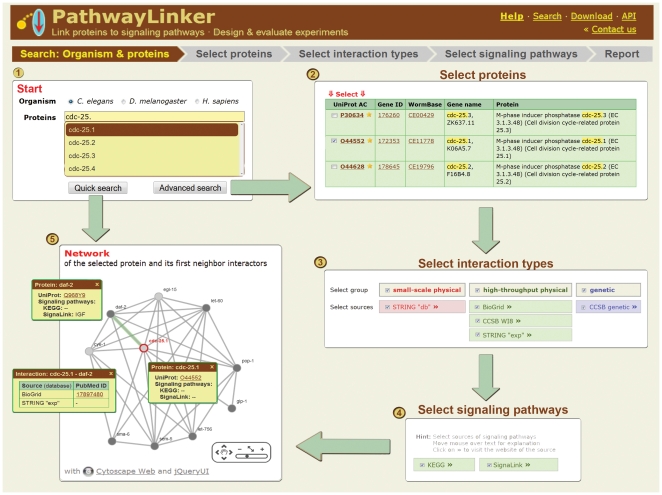
Workflow of PathwayLinker with the *C. elegans* gene *cdc-25.1* as an example.

The user should first select the species (worm, fly, or human) and enter the search terms, which can be arbitrary protein or gene names/IDs as well as drug/compound names or DrugBank IDs (Fig. 1.). Note that most genes/proteins have multiple identifiers. PathwayLinker (through the UniProt synonym search API) recognizes a large variety of name and ID types and identifies target proteins of drugs/compounds based on DrugBank. Note also the autocomplete feature: after typing a few letters, the user receives a short list of possible completions. Further typing will refine the list of suggestions. PathwayLinker has two possible search modes: quick and advanced (Fig. 1.). In a *quick search* after entering the keywords no further action is required from the user. In this case UniProt primary accessions with the highest synonym search scores for each entered search term are used automatically with together the default values of all further search parameters. In an *advanced search* the user can select

1. for each search term a set of UniProt primary accessions from the full list of accessions obtained through a UniProt synonym search for that search term;
2. sources of interactions (BioGrid, STRING, WI8, DroID, HPRD [4]–[8]) to be used for identifying first neighbor interactors, or group of these interactions (high-throughput physical, small-scale physical, genetic);
3. sources of signaling pathways (KEGG, Reactome, SignaLink [9]–[11]).

**Figure 2:**
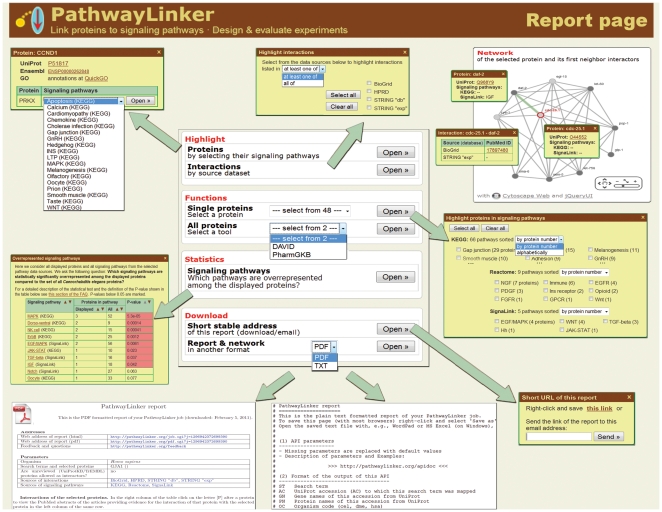
Functions of the report page of PathwayLinker. All nodes (proteins) and edges (interactions) can be clicked for further information. Proteins and interactions can be highlighted by selecting their known signaling pathways and by selecting their known interaction types, respectively. A built-in statistical enrichment test and direct hyperlinks to analyses by external resources are also available. These allow the user to select the most significantly enriched functions within the group of proteins made up of the queried protein(s) and its (their) interactors. The report is available in PDF and plain text (machine readable) formats. The user can have the stable URL of the report e-mailed to a selected address.

PathwayLinker's report page (Fig. 2.) (i) lists and interactively visualizes the network of the queried proteins and their first neighbor interactors, (ii) labels signaling pathway member proteins, (iii) provides enrichment statistics and (iv) direct links to external resources, and (v) has several download options. Among the displayed proteins, signaling pathway members can be highlighted by selecting pathway names, e.g., WNT or Notch, from a list. It is easy to select from this list, because pathways can be sorted and the number of member proteins participating in each pathway is displayed. Interactions can also be further analyzed by highlighting those present in at least one or all of the interactively marked sources (e.g., *BioGrid*, *HPRD*). A click on any displayed protein provides hyperlinks to *UniProt*, species-specific resources (*Wormbase*, *Flybase*, *Ensembl*), Gene Ontology term pages (*QuickGO*) and maps of the signaling pathways containing the given protein [12], [14]–[17]. Similarly, click on any displayed interaction to receive hyperlinks pointing to the PubMed abstract(s) of the article(s) containing experimental evidence for the interaction (literature data acquired from the selected interaction databases).

To estimate the signaling effect(s) of the queried proteins and their first neighbor interactors we provide p-values and highlight those signaling pathways significantly that are overrepresented among the displayed proteins as compared to all proteins in the given species. In addition, two external resources are available for exploring the statistically significantly overrepresented biological functions of the analyzed protein(s): *DAVID* compares protein domain structures, expression profiles and GO annotations [18], while *PharmGKB* shows pharmacological properties [19].

Currently, the PathwayLinker's report can be downloaded in two formats: plain text and PDF. Moreover, the user may return later to each report page by saving or receiving via e-mail its short stable web address (URL). For computational biologists automated programmable access is offered through an application programming interface (API). All features and downloadable files are documented in great detail under “Help” (click in the top right corner of the page).

### Examples

PathwayLinker can indicate unexpected signaling bias in normal laboratory experiments. Consider, for example, the protein interactors of the gene *cdc-25.1* (Fig. 2.). The knock-down of this gene is often used to sterilize *C. elegans* [20]: sterilized worms are used to avoid progeny in diverse experiments. We found that six of the nine first neighbor interactors of CDC-25.1 have central roles in signaling pathways (EGF/MAPK, IGF, Notch, TGF, and WNT): DAF-2 (a worm Insulin receptor), SMA-6 (a worm TGF-beta receptor), LET-60 (RAS protein), SEM-5 (GRB2 adaptor protein), POP-1 (transcription factor – TCF4), and GLP-1 (a worm NOTCH receptor). Note that all six proteins are orthologs of major human signaling proteins. Consequently, sterilizing *C. elegans* by knocking down *cdc-25.1* can globally affect the organism's signaling system and unexpectedly bias experiments.

PathwayLinker can help during the evaluation of experimentally obtained phenotypes. To predict the function(s) of a gene that has no known function so far, most genetic studies link the observed phenotypes to the phenotypes of genes with known functions and signaling pathways. This process requires a large number of interactive searches in genome and interaction databases. PathwayLinker can drastically reduce the amount of this interactive work. As an example, consider the phenotypes of the *C. elegans* gene *C27F2.4* (a predicted carboxyl methylase gene [16]), which has no known signaling pathway memberships so far. With PathwayLinker, one can find out with reduced effort that all observed loss-of-function phenotypes of *C27F2.4* are present among the phenotypes of the currently known interactors of *C27F2.4*. Therefore, the functions of these interactors and C27F2.4 possibly match. In more detail, high-throughput physical experiments [6] indicated that the protein C27F2.4 interacts with BAR-1, CLK-2 and RHA-2. Of these interactors only BAR-1, an ortholog of human Beta-catenin, is a known signaling pathway member (in the nematode WNT and EGF/MAPK signaling pathways). Since BAR-1 is a key component of the WNT pathway, we analyzed the possible relationship of *C27F2.4* with the WNT pathway in more detail. A direct link from PathwayLinker's report page points to the WormBase [16] fact sheet of *bar-1* where 3 phenotypes (slow growth, receptor mediated endocytosis defective and transgene expression pattern variance) out of the listed 14 are common knock-down phenotypes of the WNT pathway. Interestingly, WormBase lists these 3 WNT-phenotypes also in connection with the loss of *C27F2.4*; thus, *C27F2.4* is likely to function in the WNT pathway as well. Further analysis of the other 2 interactors of C27F2.4 (CLK-2 and RHA-2) showed that they do have the remaining 2 known phenotypes of *C27F2.4* suggesting its possible function in processes that involve CLK-2 and RHA-2.

PathwayLinker can also identify important signaling proteins as interactors of known human drug targets. We investigated the known drug target protein GAP JUNCTION ALPHA-1 (GJA1; also known as Connexin-43), which is currently not known to be a member of any signaling pathway. Three anti-cancer drugs – cisplatin, mercaptopurine, and methotrexate – have known effects on GJA1. [19], [21], [22]. A quick analysis with PathwayLinker (using all interaction and pathway sources) shows that GJA1 has 47 interactors that are members of 66, 9, and 5 signaling pathways according to the pathway databases KEGG, Reactome, and SignaLink, respectively. In our analysis we selected two major pathways (EGF/MAPK and WNT) that are important in development and several diseases. We found that 6 of the proteins directly interacting with GJA1 participate in the EGF/MAPK pathway (CSK, PRKA, PRKX, SRC and 2 major kinases of the MAPK pathway: ERK1 and ERK5). For the WNT pathway the analysis showed PRKA and CCND1 as direct interactors of GJA1, and 3 more key signaling proteins: CASEIN KINASE I (CSNK1D), BETA-CATENIN (CTNNB1), and a major transcription factor of the WNT pathway, LEF1. Interestingly, PRKA (Protein kinase A-anchoring protein; also known as AKAP) participates in both pathways, which increases its importance as a multi-pathway protein among the first neighbors of GJA1. Note that neither PharmGKB nor DrugBank [19], [23], two major pharmaceutical databases, list these 2 affected pathways for GJA1. In summary, for the drug target protein GJA1, that is known to be cancer-related, PathwayLinker predicts novel signaling pathway memberships. These pathway memberships can be a source of possible side effects when targeting GJA1.

## Discussion

In biomedical research modified genes/proteins often contribute to the discovery of novel molecular functions and organism-level characteristics. However, these modifications can induce unwanted side effects, often through altered signaling pathways. We have developed PathwayLinker, an online tool for estimating the possible signaling effect(s) of selected proteins and their first neighbor interactors. We have demonstrated that PathwayLinker can support the design and evaluation of experiments. First, if a gene or a protein is manipulated (e.g., by knockout or knockdown), then the developed online tool can provide a quick first estimate of how this manipulation may affect intracellular signaling and cause unwanted phenotypes. Conversely, if a protein modification (e.g., gene modification or drug treatment) causes an unexpected phenotype, then PathwayLinker can help to identify which interactor(s) and signaling pathway(s) are most likely to have contributed to that phenotype.

The recently launched online tools CentiBiN [24], NeAT [25], and MTOM [26] focus on advanced network analyses by applying, e.g., centrality measures, randomization, and topological overlap, respectively. On the other hand, PathwayLinker is more similar to DAVID [18] and BioProfiling.de [27], which focus on the functional analysis of gene lists and include signaling pathway membership and interaction data. Compared to these two, the uniqueness of the PathwayLinker lies in its ability to analyze the pathway memberships of the first neighbor interactors in a targeted way and to perform statistical tests that can evaluate even small sets of proteins (not only large gene lists).

Future plans for extending PathwayLinker are: (i) including regulatory and metabolic pathways; (ii) allowing users to upload their own pathways; and (iii) linking result sets to further external web services. The pathway and interaction databases will be updated every 12 months. Comments or suggestions are welcome. Contact information, a feedback form and detailed documentation are available at http://PathwayLinker.org.
